# Commission Government

**Published:** 1915-01

**Authors:** 


### Commission Government.

We would not have felt justified in discussing this subject so long as it remained a political issue. But, as a comparatively new policy in municipal government, actually adopted by Buffalo and Niagara Falls, two important cities in our territory, we feel that it deserves some consideration, even in a medical journal. First of all, whatever may be said as to the theoretic desirability and the practical conflicts of political and medical careers, united in the same person, the physician should be a citizen not only in the passive sense but in the active sense of possessing judgement and influence commensurate with his education and the opportunities for developing sagacity and broadness of view in his daily experience. Secondly, the physician should normally belong to the class which feels directly or only one step removed, the burdens of citizenship. Thirdly, municipal conduct involves, not only in the Dept. of Health but in various other branches, notably those dealing with water, sewage, garbage and waste, maintenance of streets, education, poor relief, etc., problems on which the judgement of the medical profession as a whole, should have a very direct influence.

In no spirit of partisanship, it should be clearly understood that the mere change of method of administering municipal affairs does not in any way eliminate the necessity of watchfulness against the usurpation of delegated power to personal or political ends. It was neither intended nor expected that the representative and executive elective system of government should be thus abused and, conversely, let us not be so optimistic as to believe that the new tools of government are incapable of being put to uses to which they are not intended. We may even go so far as to point out that the division of responsibility among several men entrusted with the general supervision of quite diverse lines of work, with whose details they cannot be familiar, involves its own dangers which must be carefully weighed by actual experience against the dangers of a subdivision of individual authority theoretically, at least, implying special fitness for the greater or less degree of executive responsibility involved. The selection of a commission form of government represents a deliberate judgement of the people and a sincere desire for reform. This fact, as well as the necessity of learning how to use new political tools for political and personal purposes, almost guarantees an enormous initial advantage but each citizen must realize that only his continual attention to his duties will prevent the ultimate breach of trust which has led to the downfall of the former system of government.

It should also be realized that there was an element of sound sense in the former recognition of local representation within the confines of a single municipality. This element should be recognized as identical in theory with the principal of—in the main—home rule for the city as a whole, or local representation of various districts in the state or national government. The broad interests of the nation, state or city, should not be subordinated to unworthy local selfishness but, on the other hand, the existence of marked differences in needs and in the means for gratifying them in various localities of a city, is an actual fact which must not be ignored. Neither should any part of a city be distinctly a benefactor nor a beneficiary at the hands of any other part, so far as can be practically avoided. The alderman of a ward has a local interest and pride and a familiarity with local conditions which may be used for both good and bad ends. The commissioners of the whole city must realize the need of familiarizing themselves with local needs.

Citizens can take an active and practical part in municipal government only when provision is made for encouraging them in contributing such information as they may have and for insuring respectful consideration not only in the personal treatment of the complainant but in attention to the suggestion. Obviously, Citizen Tom may be influenced by personal interests Citizen Dick may be a crank, but Citizen Harry may really call attention to an unnecessary waste or to an opportunity to save a good deal of money by promptly attending to something out of order. For instance; streets have repeatedly been entirely repaved because none of the citizens using them, to their own inconvenience and danger, have had the power to start repair work in time. Recently, we have heard of a disinfectant sold to the city at a very liberal gross profit, some of which is accidentally left on the desks of officials. A lady leaving a document for filing was charged 90 cents; she said she had been told the charge would be 50 cents; and the clerk compromised on 75. This was of course, at the County Clerk's office, but the principle is the same, especially when we reflect that every good sized city should be a county if not a state. A few years ago, a school principal saved the city several thousand dollars on a school site and the officials and dealers who were interested in another deal actually took steps toward his removal.

Aside from prompt attention to repairs and individual instances of inefficiency and graft, the Commissioners should clearly comprehend that their main task is to reduce the cost of the city government. Buffalo has excellent school, fire, police and health departments. Unless figures lie, it has the most liberal supply of water in the world and, allowing for the apparent fact that at least half of its relatively low typhoid rate is due to imported cases or imported infection, it has nearly the best water in the world, short of sand-filtration or specially guarded water sheds. It has, absolutely, more smooth pavement than any other city in the world, irrespective of population, unless according to very recent statistics and, to the best of our knowledge, since the completion of the work on South Elmwood avenue, it has no unpaved street except in the outskirts where the demand has not yet occurred. It has ample Park room, except for the need of a few more small local breathing spaces and play grounds.

The streets are well lighted—except from the standpoint of speeders or those who think that business streets should resemble an amusement park and that residence streets should have no night. They are well cleaned. We do not overlook the opportunity that these statements open to pessimists to cite exceptions and to make complaints, but we feel that the statements are true in the broad sense and that the exceptions are just such as require the close touch between city officials and private citizens already mentioned.

The present mayor, even when a candidate for re-election, when promises of economy offer a specious advantage, came out flatfootedly with the opinion that a city cannot maintain the standards of excellence demanded under present conditions, on a low taxation. One of the assessors, in reply to a protest against a tax which had virtually amounted to a confiscation of one-sixth of the cost of a piece of property, stated that a low tax-rate was simply a matter of juggling, that the rate could not be lowered except by raising assessed values or discovering more taxable personal property. The tax-payer who does not use the public schools for his children, is contributing, under compulsion, to charity of one form or another, to the extent of at least 25 per cent. of his entire tax. Omitting the cost of public instruction, at least a tenth of the remainder of the tax is distinctly a matter of charity—not meaning by this an average share of general protection against crime or disease but money actually paid for the support or comfort of a dependent class of one kind or another. To illustrate how municipal expenses increase in a very laudable way: in 1880, Buffalo had a population of about 155,000, just about a third of its present population; the high school graduates then numbered 30 to 40 a year; now (1914) 525, approximately 5 times as many proportionately to the increase in population. An additional year has been added which may be considered either as the beginning of the high school course or the end of the grammar school course, and applying not to the 525 graduates but to about ten times as many pupils.

In various ways, taxes have increased because of increased duties assumed by the municipality. In striving for economy, the Commissioners should not—with some few exceptions—reduce the burdens of the city. Neither should they represent the reform movement to the degree of assuming all sorts of fancy devices for philanthropy, advocated by a certain type of reformer. In passing, it may be noted that the professional graftsman, seeking only his own welfare, however dishonestly, is not so dangerous from the economic standpoint as this type of reformer who spends an abundance of leisure devising schemes for spending one man's money for the benefit, more or less real, of another. We are strongly inclined to the opinion that any reduction in municipal expenses must come from careful attention to details.

